# Investigating the Durability of PHA-Coated Burlap for Coastal Restoration

**DOI:** 10.1021/acsaenm.6c00063

**Published:** 2026-03-31

**Authors:** Roya Gadimli, Claire Thomas, Melissa M. Omand, Julie N. L. Albert

**Affiliations:** † Chemical and Biomolecular Engineering, 5783 Tulane University, New Orleans, Louisiana 70118, United States; ‡ Graduate School of Oceanography, 4260 University of Rhode Island, Kingston, Rhode Island 02881, United States

**Keywords:** Polyhydroxyalkanoates, Biodegradable coatings, Marine degradation, Coastal restoration materials, Burlap reinforcement

## Abstract

Many synthetic textiles are used in coastal restoration because natural fiber materials have lower durability and may degrade too quickly; however, these synthetic materials pose environmental risks, such as microplastic pollution. Motivated by the need for materials that balance durability with environmental sustainability, we assessed the feasibility of polyhydroxyalkanoate (PHA) coatings as a strategy to prolong the service life of burlap fabric. Three PHA-based polymers were applied by dip-coating, single-sided melt pressing, and double-sided melt pressing and then immersed in seawater at ∼5 m depth for 288 days. Visual observations, evaluation of the end-point mass loss data, and complementary molecular weight and X-ray scattering analyses show that durability depends on the processing method and polymer crystallinity: double-sided melt-pressed samples retained the most mass (compared to single-sided, dip-coated, and uncoated), and mass loss decreased with increasing initial PHA polymer crystallinity. Additionally, these data support a degradation pathway in which amorphous domains are preferentially eroded and crystalline lamellae thicken due to relaxation of entanglement constraints. These processing–crystallinity–durability relationships suggest that the residence time may be tuned to match restoration schedules, positioning melt-pressed PHA coatings as an ecologically sustainable alternative to persistent plastic materials.

## Graphical abstract

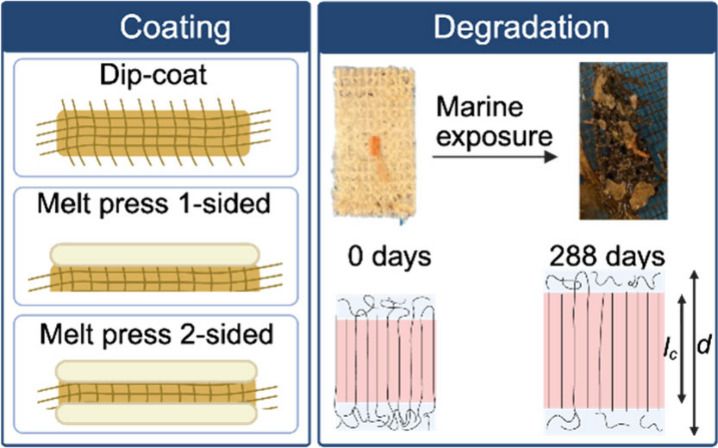

## 1 Introduction

Coastal erosion removes more than 80,000 acres of land annually in the United States, endangering the country’s sandy shorelines and incurring direct damages in excess of $500 million per year.^1^ These losses are expected to escalate as sea-level rise and storm intensification amplify wave energy and shoreline retreat, making the development of ecologically sustainable protection strategies a global priority.

Synthetic materials, such as plastic-based geotextiles, silt fences, and turf reinforcement mats are widely used in erosion control^2^ due to their durability, but these materials pose long-term environmental risks, including microplastic pollution and habitat disruption.^3–6^ In contrast, natural materials, such as jute, flax, cotton, and coir, offer an environmentally friendly alternative to synthetic geotextiles because they are biodegradable and allow for plant growth and sediment retention.^5,7^ However, despite these advantages, natural materials degrade rapidly in marine environments, often disintegrating before they can fully serve their intended function. This premature breakdown compromises their ability to serve as a protective barrier and retain sand for shoreline replenishment and reinforcement.

Thus, the trade-off between longevity and biodegradability has been a central issue in developing effective coastal restoration materials,^7^ necessitating research into solutions that balance durability with environmental sustainability. One promising research direction involves the use of biodegradable polymer coatings to extend the lifespan of natural-fiber-based erosion control materials. Among these, polyhydroxyalkanoates (PHAs) have emerged as a potential solution due to their marine biodegradability and environmental friendliness. PHAs are biopolyesters produced by microbial fermentation, offering hydrophobicity, mechanical stability, and tunable degradation rates. Although PHA degradation in the environment releases carbon dioxide (aerobic conditions) or methane (anaerobic conditions), they also can be synthesized via microbial fermentation, which consumes carbon dioxide and can be accomplished using waste carbon feedstocks (e.g., food waste, wastewater, and agricultural byproducts).^8–12^ PHAs have been extensively studied in biomedical and packaging applications,^10,13–16^ but their potential for integration into environmental restoration materials remains largely unexplored.

This study aims to evaluate the effectiveness of PHA coatings to enhance the durability of burlap fabric in a marine environment. Three distinct PHA polymers were tested by using different coating techniques, including dip-coating and melt-pressing methods. The primary objectives were to determine how these coatings influenced the degradation rate of burlap and to assess the relationship between polymer crystallinity and structural integrity. The degradation mechanism was also investigated by comparing molecular weight distributions and features of the crystal structure before and after exposure to marine conditions.

## 2 Materials and Methods

### 2.1 Materials

Three polyhydroxyalkanoate (PHA) polymers were utilized in this study: plant-based poly­(3-hydroxybutyrate) (P3HB) powder provided by Mango Materials (P1), injection-molding-grade YOPP pellets compounded by Mango Materials (P2), and TLREEFS PHA pellets (P3) purchased from Amazon. P3 was identified as mainly poly­(3-hydroxybutyrate-*co*-3-hydroxyvalerate). However, additional details on the P2 and P3 compositions are proprietary and either subject to confidentiality agreement (Mango Materials) or not disclosed by the supplier (TLREEFS). Commercially available untreated burlap fabric, reclaimed from local coffee companies, was cut into rectangular samples (5 cm × 4 cm) for coating and subsequent testing. The burlap fabric exhibited an areal density of 313 ± 17 g m^–2^ and was identified as jute-based (see the Supporting Information). Chloroform (analytical grade) was used as a solvent in the dip-coating application.

### 2.2 Coating

Three coating techniques were applied to the burlap samples. For the dip-coating method, burlap samples were fully submerged in a 4% (w/v) PHA solution in chloroform. Excess polymer solution was drained, and samples were air-dried at room temperature for 24 h. In the single-sided melt-press method, PHA pellets were melted between Teflon sheets on a hot plate at 190 °C, pressed by hand for ∼1 min to form a thin film, and then removed from the hot plate to cool. The top Teflon sheet was removed and replaced with a burlap sample, which was then placed back on the hot plate, repressed, and cooled again to produce a single-sided coating on the burlap. The double-sided melt-press method followed the same procedure as the single-sided method, but the burlap was sandwiched between two polymer films to achieve a coating on both sides. A total of nine coated samples (3 coating methods × 3 polymers) were prepared. A subset of these samples was selected for environmental testing to enable direct comparisons between the coating methods and polymer types. All coated burlap samples were trimmed to 2.5 cm × 4 cm for environmental testing.

### 2.3 Environmental Testing

Coated and uncoated burlap samples (one sample per condition due to space limitations) were deployed in a fish exclusion canister submerged approximately 5 m below the ocean surface at the University of Rhode Island GSO Dock (41.492072 and −71.419117). The setup exposed the samples to natural coastal conditions, including temperature fluctuations, salinity variations, and microbial activity. Sample degradation was assessed qualitatively each month using photographic documentation and descriptive notes to track structural integrity and visual degradation trends. After 288 days of immersion, each sample was gently rinsed to remove salts and debris and then dried in air. To quantify degradation, the initial and final masses of each sample were measured using an analytical balance before and after 10 months of immersion in the marine environment. Next, visible polymer fragments were gently peeled with tweezers. The samples were then rinsed with chloroform to separate coating from burlap fibers. See Figures S3 and S4 for photographs of the environmental testing setup.

### 2.4 Crystallinity and Lamellar Long Period Analysis

Crystallinity analysis was conducted by small- and wide-angle X-ray scattering (SAXS and WAXS) using an Anton Paar SAXSpace instrument operated in line-focus mode (40 kV and 50 mA) with Cu Kα radiation (λ = 0.15418 nm). Data were collected in transmission with a fully evacuated beam path to suppress air scatter. The measured scattering-vector range was *q* = 0.1–18 nm^–1^, spanning SAXS (microstructure/long period) and WAXS (crystalline lattice). The lamellar long period (*d*) was determined from the SAXS peak position by using *d* = 2π/q*, where q* corresponds to the primary scattering peak. This assignment is consistent with the well-established scattering behavior of semicrystalline PHAs and related polyesters, in which alternating crystalline and amorphous regions produce a first-order Bragg reflection.^17,18^ WAXS data were processed to determine the crystalline and amorphous peak areas from which the degree of crystallinity, *X*_c_(%), was calculated (Figure S5 and Table S2). The crystalline and amorphous contributions to the WAXS profile were resolved by peak fitting, using the known scattering pattern of P3HB as a reference^19^ (Figure S6). For SAXS/WAXS analyses, PHA was isolated from burlap by mechanically removing it from the fabric.

### 2.5 Molecular Weight Measurements

A portion of the sample recovered for SAXS/WAXS analysis was subsequently dissolved in chloroform for gel permeation chromatography (GPC) analysis. Number-average (*M*_n_) and weight-average (*M*_w_) molecular weights were determined by GPC on a Waters e2695 HPLC platform equipped with a refractive-index detector. Analyses were conducted at 30 °C in chloroform (flow rate of 0.3 mL min^–1^). The instrument was calibrated with narrow-dispersity polystyrene standards; the resulting polystyrene-equivalent molar masses were converted to PHA molar masses by applying the appropriate Mark–Houwink–Sakurada parameters (*K* and α), as detailed in Table S1. (Note: To confirm the reliability of peak fits, chromatograms were fit with different peak widths, initial guesses, and intensities, and it was verified that they resulted in comparable molecular weight distributions.)

## 3 Results and Discussion

### 3.1 Visual Observations and Mass Loss Trends

The study experimented with three coating methods—dip-coating, single-sided melt press, and double-sided melt press—using three different PHA polymers: P1, P2, and P3. A subset of the nine possible coating–polymer combinations was selected for environmental testing to enable direct comparisons between the coating methods and polymer types. Specifically, P1 dip-coated and single-sided melt-press samples enabled a comparison of dip-coating and melt-pressing methods, single-sided versus double-sided melt pressing was evaluated for P2 and P3, and all polymers were compared with the single-sided melt-press preparation. This design prioritized a systematic comparison within the physical constraints of a single canister deployment. Photographs of the as-prepared samples (Figure 1, 0 days) show that dip-coating deposited polymer on the burlap threads but did not fill holes in the weave; in contrast, both melt-press methods filled the holes in the weave with polymer. Subsequent photographs taken at regular intervals over the study period (Figure 1, 155–288 days) show that uncoated and dip-coated samples frayed and deteriorated within 265 days (∼9 months). We interpret this as disintegration/dispersion rather than confirmed degradation, in line with reports that cellulose fiber biodegrades within ∼1 month^20^ in marine waters and that jute twines typically lose integrity over ∼4–8 months.^21^ Samples with a single-sided melt-press coating exhibited an intermediate durability, staying mostly intact for the first 265 days but degrading substantially by 288 days (∼10 months). In contrast, samples with a double-sided coating remained visually intact for the duration of the experiment (288 days and ∼10 months). These images also display the progressive deterioration of the burlap weave, including widening of holes, fraying and loosening around edges, and disruption of weave alignment, with the severity of these features depending on the extent of polymer protection provided by each coating method.

**Figure 1:**
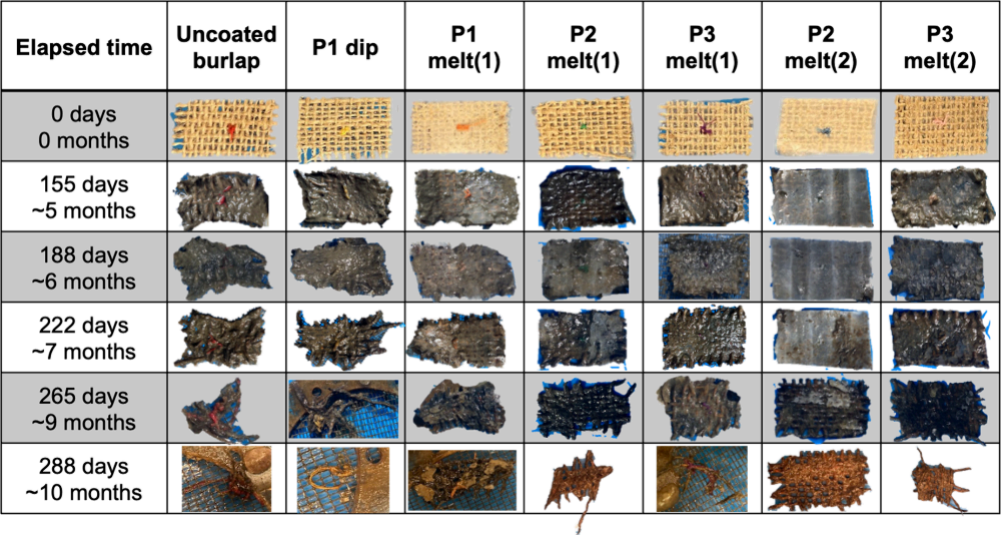
Photographs of 2.5 cm × 4 cm burlap samples taken over 288 days (10 months) of immersion in the marine coastal environment: no polymer (uncoated burlap); P1 (dip-coated and single-sided melt-pressed); P2 (single- and double-sided melt-pressed); P3 (single- and double-sided melt-pressed). Images at 0, 155, 188, 222, 265, and 288 days show progressive weave fraying, hole widening, and edge unraveling. Fiber loss becomes apparent starting at 222 days.

Mass loss analysis provided a quantitative measurement of the degradation of each sample. In Figure 2, each bar shows the sample mass divided into “burlap” and “polymer” contributions before and after environmental exposure, with annotations above each bar noting the percent polymer in the sample. The initial and final masses after 10 months revealed significant differences among the coating types and polymers. Specifically, the dip-coated (P1 dip) sample was fully degraded. The single-sided melt-press coated samples [P1 melt(1), P2 melt(1), and P3 melt(1)] exhibited moderate degradation, with P1 showing the least mass loss, while P3 degraded completely. The double-sided melt-press coated samples [P2 melt(2) and P3 melt(2)] were most effective for protecting the burlap, resulting in minimal mass loss and demonstrating strong resistance to environmental degradation. In all samples, it was primarily the polymer layer that degraded, visibly and quantitatively protecting the underlying burlap fabric.

**Figure 2:**
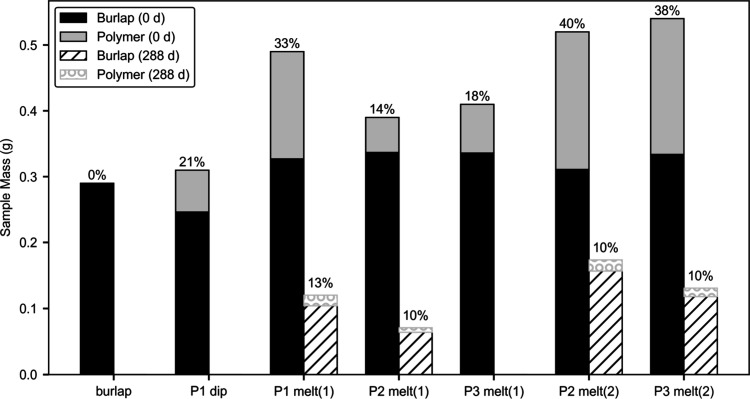
Initial mass and residual mass after 288 days of each sample. Stacked bars represent the burlap and polymer compositions of each sample, and the annotations above the bars report the mass percentage of polymer in the sample. Complete sample loss was observed for burlap, P1 dip-coated (P1 dip), and P3 single-sided melt-pressed [P3 melt(1)].

### 3.2 Crystallinity and Degradation

To explain the differences in degradation between polymers P1, P2, and P3 seen for the same coating method, the initial degree of crystallinity was identified as a correlating feature, regardless of the PHA polymer formulation (Figures S3 and 3). In general, higher crystallinity values correlated with a lower mass loss over the experimental period.

**Figure 3:**
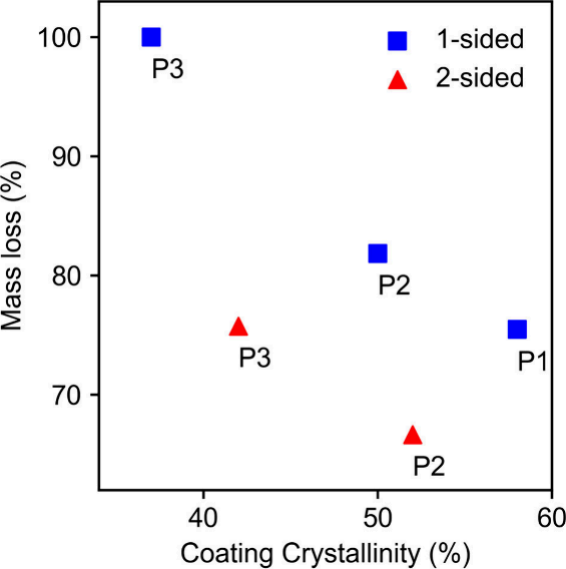
Relationship between the initial PHA coating crystallinity and composite (burlap + PHA) mass loss after 288 days. Single-sided (squares) and double-sided (triangles) coatings both show lower mass loss at a higher initial crystallinity.

This trend is well-documented in the PHA literature, where crystalline domains are known to reduce chain mobility and slow enzymatic cleavage.^17,22,23^ Relevant to both one- and two-sided samples, the more crystalline P2 coatings withstood marine exposure better than P3, exhibiting markedly lower mass loss. Although some reports suggest that the correlation between crystallinity and degradation is not always linear and can be influenced by other parameters, such as the lamellar thickness, microstructure, and environmental temperature gradients,^18^ our end point data clearly show that higher crystallinity PHA variants protected burlap fibers more effectively. Additionally, SAXS analysis of melt-pressed polymer samples at *t* = 0 reveals comparable lamellar thicknesses across all three polymers (3.5–3.9 nm), with no systematic difference detected as a function of polymer identity or processing method (Figure S7), further supporting crystallinity as the dominant structural parameter controlling degradation resistance in this study.

### 3.3 Degradation Mechanisms

#### 3.3.1 Macroscale Mechanisms

Based on qualitative observations made during sample handling prior to environmental exposure, differences in mechanical behavior were noted even before immersion (0 days): single- and double-sided melt-pressed specimens were noticeably stiffer and more cohesive than both uncoated and dip-coated samples. Additionally, the melt-pressed films bonded intersecting yarns, restrained loose ends, and stuck frayed fibers together; in contrast, dip-coated samples were only slightly stiffer than uncoated burlap, and individual filaments remained free to spread. During immersion, single-sided melt pressing was superior to dip coating primarily because the continuous polymer film covered one entire face of the 2.5 cm × 4 cm specimen, effectively halving the surface area of burlap directly exposed to seawater. This reduction in accessible area would have slowed moisture uptake and enzymatic attack^24^ compared to dip-coated samples. Nevertheless, the uncoated reverse face of the single-sided specimens continued to absorb water, swell, and fray, allowing degradation to propagate from that side and producing a moderate long-term performance. Double-sided melt pressing offered the most uniform barrier by covering both faces of the fabric with a continuous PHA layer.

#### 3.3.2 Adhesive/Microscale Mechanisms

Beyond the macroscale barrier effect, the melt-pressed coatings also contributed to sample integrity at the microscale by adhering to the fiber network so that, even when holes formed in localized regions, the surrounding polymer held the weave together and prevented the separation of fibers. This adhesive effect explains why P2 single-sided melt-pressed samples, despite containing less polymer and exhibiting lower crystallinity than P1 dip-coated samples, still provided superior protection. Thus, durability emerged not only from coating coverage and crystallinity but also from how the polymer mechanically coupled to the fiber architecture to suppress unraveling of the fabric.

#### 3.3.3 Polymer Microstructure and Molecular Mechanisms

Although more frequent sampling and characterization at intermediate time points would yield insights into the real-time evolution of mass loss and polymer morphology during degradation, this type of investigation was beyond the current study scope due to limits in the number of samples that could be deployed simultaneously and challenges associated with characterization in the marine environment. However, we can derive insight into the probable polymer degradation mechanism by comparing start- and end-point data interpreted within the context of the broader PHA literature.

The correlation between crystallinity and mass loss suggests preferential microbial attack on amorphous regions, which has been seen in other biodegradable polymer systems.^25–27^ Closer examination of the molecular weight and structural changes of P1 (chosen because it is additive-free) from the start (0 days) to the end (288 days) is consistent with this type of degradation mechanism.

In this mechanism, targeted erosion of the less-ordered amorphous matrix both shortens the average chain length and simultaneously releases lamellar stacks from spatial constraints, allowing them to thicken (Figure 4). These changes result in a decrease in the molecular weight dispersity (GPC, Figure 5a), an extension of the lamellar long period (SAXS, Figure 5b), and a rise in crystallinity (WAXS, Figure 5c). Quantitative analysis of these changes further supports the mechanism proposed in Figure 4.

**Figure 4:**
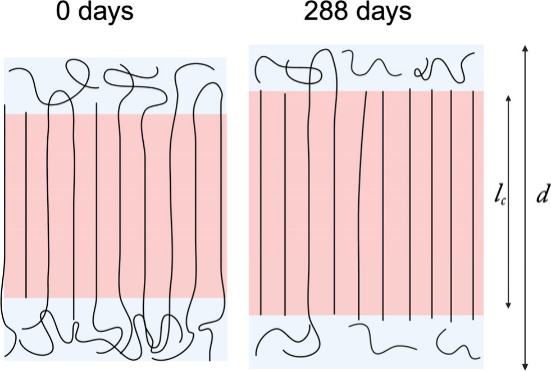
Schematic of the molecular degradation pathway proposed for P1. Preferential erosion of amorphous domains removes folds and reduces entanglements, enabling lamellar thickening in the remaining material. *d* and *l*_c_ stand for a long period and the lamellar thickness, respectively.

**Figure 5:**
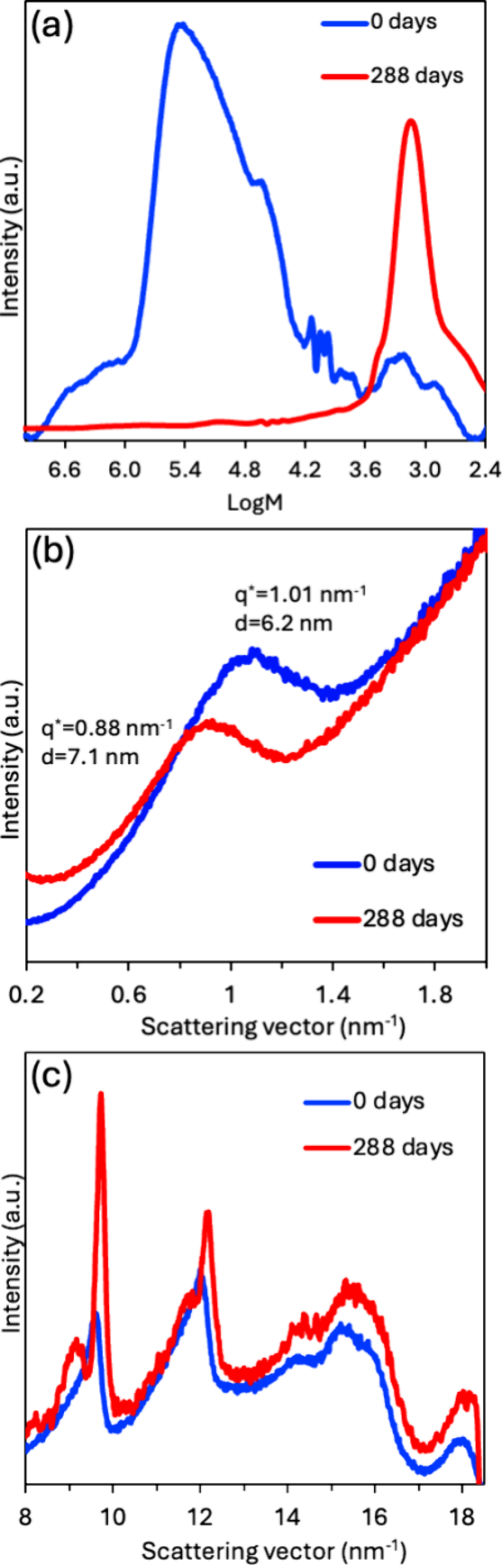
Molecular weight distribution and structural evolution of P1 over 288 days. (a) GPC traces show a narrowing of the molecular weight distribution and a loss of high-molecular-weight chains. (b) SAXS profiles indicate an increase in the long period from 6.2 to 7.1 nm. (c) WAXS patterns exhibit sharper diffraction peaks and increased crystalline peak area.

Specifically, GPC chromatograms reveal a pronounced narrowing of the molecular weight distribution during the 288-day degradation period. At 0 days, P1 exhibits an ultrabroad GPC distribution. A dominant peak centered near log *M* ∼ 5.4 (corresponding to 280 kDa) represents the bulk of the material, yet the smaller set of peaks around log *M* ∼ 3.2 (2 kDa) serves a crucial role architecturally. Chains in this 10–20 repeat-unit window are just long enough to crystallize as fully extended stems and therefore dictate the initial lamellar thickness. To monitor the changes in the chains guiding the lamellar thickness, we deconvoluted this section of the chromatogram (0.3–4.7 kDa) into three Gaussian modes (Figure 6a). We see that this molecular weight range also corresponds to the primary peak that remains after degradation (Figure 6b), with the trace consisting of a well-defined peak (log *M* ∼ 3.2) and near disappearance of the highest- and lowest-molecular-weight chains. These changes in molecular weight distribution confirm extensive backbone scission during environmental exposure.

**Figure 6:**
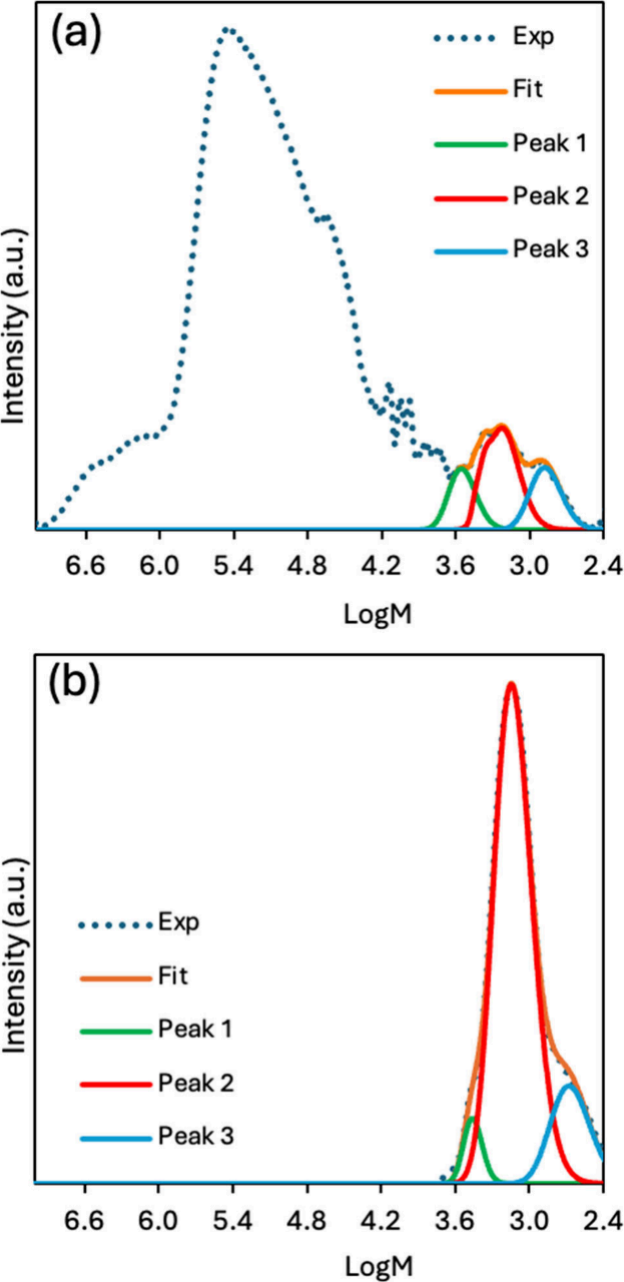
Gaussian deconvolution of P1 GPC chromatograms at (a) 0 and (b) 288 days into three partially overlapping peaks. After degradation, Peak 2 dominates, with the relative fractions of Peaks 1 and 3 diminishing.

Concurrently, SAXS shows that the long period, *d*, increases from 6.2 to 7.1 nm after 288 days of degradation (Figure 5b). When the long period (*d*) obtained from SAXS is combined with the crystallinity (*X*_c_) derived from WAXS, the lamellar thickness (*l*_c_) can be calculated according to *l*_c_ = *d* *X*_c_. Like *d*, the lamellar thickness (*l*_c_) also increases, from 3.6 to 4.9 nm, while the thickness of the amorphous region (*l*_a_ = *d* – *l*_c_) decreases from 2.6 to 2.2 nm.

This result appears counterintuitive when viewed alongside the GPC data, which points to a substantial loss of high-molecular-weight material. However, the apparent paradox can be resolved by considering how specific chain lengths contribute to lamellar assembly.

The increase in *l*_c_ can be explained by considering how different length chains are incorporated during crystallization. First, short, fully extended chains (∼1–2 kDa based on GPC) define the upper limit of the lamellar thickness. Sufficiently long chains may form folds or bridges to accommodate this constraint.^28^ Intermediate length chains will crystallize up to the lamellar thickness defined by the short chains, relegating the remaining chain length to the amorphous region.^29^ During the initial film formation, all chain lengths would crystallize simultaneously, and chain entanglements in the amorphous region would have limited lamellar thickening. Degradation of long chain segments in the amorphous region during environmental exposure releases the constraints imposed by chain entanglements, folds, and bridges, which allows lamellar thickening to occur and results in an increase in *l*_c_. This interpretation aligns with the findings of Androsch et al.,^30^ who showed that P3HB chains with low *M*_n_ organize as fully extended rods incapable of folded conformations, effectively dictating the initial lamellar dimension. Consistent with the increase in *l*_c_, WAXS patterns show sharper, more intense diffraction peaks after degradation, indicating an improved crystalline order in the residual material.

To translate the SAXS/WAXS observations into molecular metrics, we calculated the degree of polymerization corresponding to the lamellar thickness according to DP = *l*_c_/*x*_0_, where *x*_0_ is the axial length of a single repeat unit. Adopting the crystallographic conventions of Phongtamrug and Tashiro^19,31^ for P3HB, *x*_0_ was taken as half of the *c* axis for the α form (i.e., *x*_0,α_ = 0.5 × 0.0596 nm = 0.298 nm) and the full *c* axis for the β form (i.e., *x*_0,β_ = 0.466 nm). With ∼20% β content (see the Supporting Information), the weighted average gives *x*_0_ = 0.332 nm. The β form is atypical for nondrawn films, but processing-induced stretching could have produced β crystals, as noted by Mottin et al.^32^ From these calculations, we find that DP corresponding to *l*_c_ increases from 11–12 to 15–16 from 0 to 288 days (Table 1).

**Table 1:** Comparison of Structural Parameters and Molecular Weight for P1 at 0 and 288 days: SAXS Long Period (*d*), Lamellar Thickness (*l*_c_), DP_n_ Derived from SAXS (DP_n,SAXS_), DP_n_ Derived from Deconvoluted GPC Peaks (DP_n,GPC_), and the Weight Percent Contribution of Each GPC Peak to the Distribution^a^

	0 days	288 days
*d*	6.2	7.1
*l*_c_	3.6	4.9
DP_n,SAXS_	11–12	15–16
DP_n,GPC_ (wt %)		
Peak 1	31–45 (29%)	26–38 (7%)
Peak 2	22–33 (50%)	12–18 (81%)
Peak 3	6–10 (21%)	4–6 (12%)

a Comparison of the DP_n,SAXS_ and DP_n,GPC_ values supports the hypothesis that shorter, full-extended chains dictate the lamellar thickness.

For comparison, GPC chromatograms can be fit to determine the approximate DPs for subpopulations of chains present in a sample. At *t* = 0 days, the GPC chromatogram of P1 (Figure 6a) resolved into three partially overlapping peaks spanning *M*_n_ ≈ 0.3–4.7 kDa (number-average DP, DP_n_ = 3–55). Peak 2 (DP_n,2_ = 22–33) dominated, contributing 50 wt % of the polymer, Peak 1 (DP_n,1_ = 31–45) accounted for 29 wt %, and Peak 3 (DP_n,3_ = 6–10) made up the remaining 21 wt %. The degree of polymerization calculated from the SAXS long period (DP = 11–12) lies between DP_n,2_ and DP_n,3_ (Table 1). Because chains of this length are long enough to crystallize without folding, they set an upper bound on the lamellar thickness and effectively “pin” the long period in the original film. Chains belonging to higher-mass peaks adopt folded conformations or bridge multiple lamellae to fit within the crystal lattice, whereas lower-mass chains, which are too short to span the full lamellar thickness, are likely excluded to the amorphous regions.^33^

After 288 days of marine exposure, the molecular weight distribution contracted to three modes (Figure 6b), which correspond to Peaks 1–3 of the 0-day chromatogram. Specifically, Peak 2 narrowed, shifting DP_n,2_ to lower values (DP_n,2_ = 12–18), and became dominant, contributing 81 wt % of the remaining polymer. This DP corresponds to the 15–17 repeat units obtained from the 288-day lamellar thickness (Table 1). The high-DP fraction (Peak 1) diminished to 7 wt %, while small chains shorter than the lamellar span (Peak 3, DP 4–6) comprised only 12 wt %. This convergence on a narrow DP range is consistent with selective hydrolysis of ester bonds in amorphous domains, which proceeds more rapidly than in densely packed crystalline regions.^23,34^ Preferential cleavage of tie-chains and fold segments of longer chains depletes the high-mass tail, while short chains relegated to the amorphous region can diffuse out. In contrast, chain stems already locked into lamellae are sterically shielded and persist, ultimately dominating the molecular weight distribution.

## 4 Conclusions

This work demonstrates that PHA coatings transform rapidly biodegradable burlap into durable yet fully biodegradable material. Of the coating methods examined, double-sided melt-pressed samples preserved the greatest fraction of burlap mass over 288 days of marine exposure, whereas uncoated or dip-coated counterparts disintegrated within 9 months. The enhanced persistence correlates with the polymer crystallinity (i.e., lower mass loss for higher crystallinity coatings) and area of burlap exposed to the environment (i.e., double-sided offered better protection than single-sided or dip-coated).

Combined GPC–SAXS–WAXS analyses support the conclusion that PHA degradation is governed by a selective erosion mechanism in which hydrolytic and microbial attacks preferentially target amorphous domains, narrowing the molecular weight distribution and eliminating chains that bridge or fold between lamellae. The released chains reorganize into thicker crystalline stacks, thereby increasing the lamellar thickness. As reported by Koike et al.,^22^ lamellar thickening slows degradation of the PHA polymer; for the coated materials studied here, this phenomenon would extend the time PHA coatings and can protect the burlap fabric.

Taken together, these findings position melt-pressed PHA films as tunable coatings to reinforce natural fiber materials. By adjustment of the film thickness,^35^ coating method, and crystallinity, the functional lifetime of these materials could be matched to specific environmental restoration schedules.^36^ Thus, this work provides insights into erosion-control materials that balance longevity with ecological responsibility.

## Supplementary Material



## Notes

Fourier transform infrared and WAXS characterization confirming the burlap fiber identity as jute, photographs of the coastal immersion setup and sample retrieval, and details of GPC molecular weight calculations and WAXS crystallinity fitting (PDF)
