# Supplementary material for: Investigating the Durability of PHA-Coated Burlap for Coastal Restoration
Source: ACS Appl Eng Mater. 2026 Mar 31;4(4):1795–801. doi: 10.1021/acsaenm.6c00063 (PMC13126563; doi:10.1021/acsaenm.6c00063)
Supplement: Supplementary file 1 [file em6c00063_si_001.pdf]

Supporting Information

Investigating the Durability of PHA-Coated Burlap for Coastal Restoration

Roya Gadimli,[1] Claire Thomas,[2] Melissa M. Omand,[2] Julie N. L. Albert[1*]
[1]Chemical and Biomolecular Engineering, Tulane University, New Orleans, LA 70118
[2]Graduate School of Oceanography, University of Rhode Island, Kingston, RI 02881
[*]Corresponding Author, Email: jalbert6@tulane.edu

Contents

Burlap Characterization S2
Figure S1. FTIR spectra of burlap fibers showing characteristic lignocellulosic bands consistent with jute. S2
Figure S2. Wide-angle x-ray scattering (WAXS) patterns of burlap fibers showing crystalline reflections consistent with jute. S2
Setup for Immersion in Marine Coastal Environment S3
Figure S3. Photographs of the environmental testing setup. S3
Figure S4. Example of monthly sample retrieval. S4
GPC Fitting S5
Table S1. Number-average molecular weights (M_n) obtained for each Gaussian-deconvoluted GPC peak S5
WAXS Fitting S6
Figure S5. Representative WAXS curve fitting S6
Table S2. Degree of crystallinity of polymer coatings (P1, P2, P3) determined from WAXS. S6
Figure S6. Identification of peaks in WAXS patterns of P1 .... S7
SAXS Analysis S8
Figure S7. SAXS analysis of melt-pressed polymer samples at t = 0. .... S8
References S9

Burlap Characterization

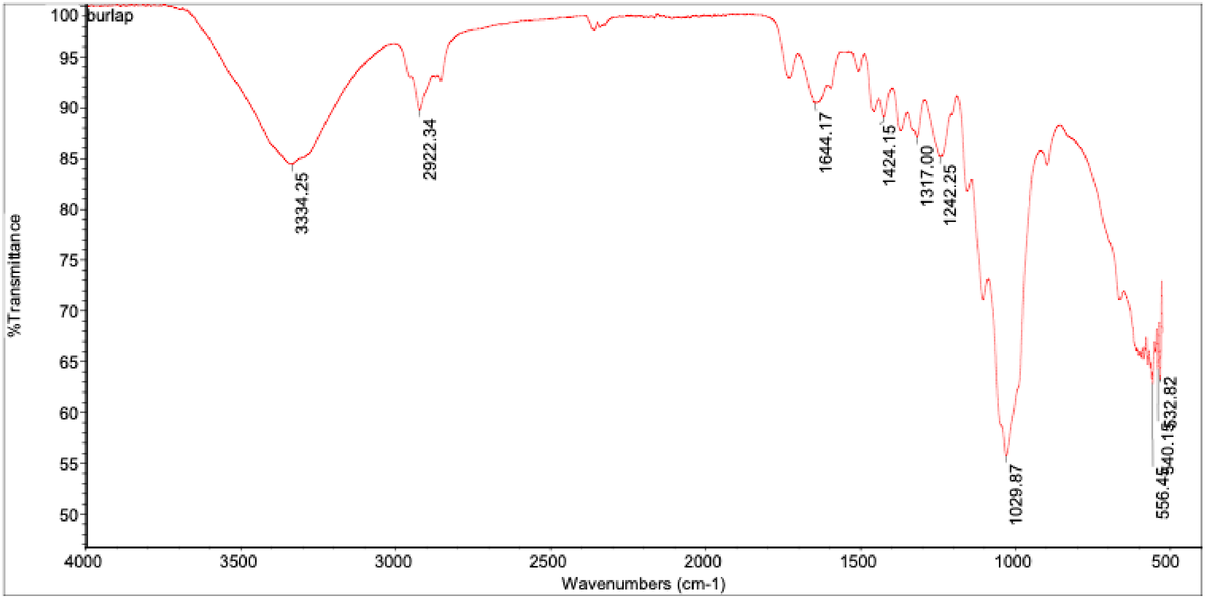

Figure S1. FTIR spectra of burlap fibers showing characteristic lignocellulosic bands consistent with jute.

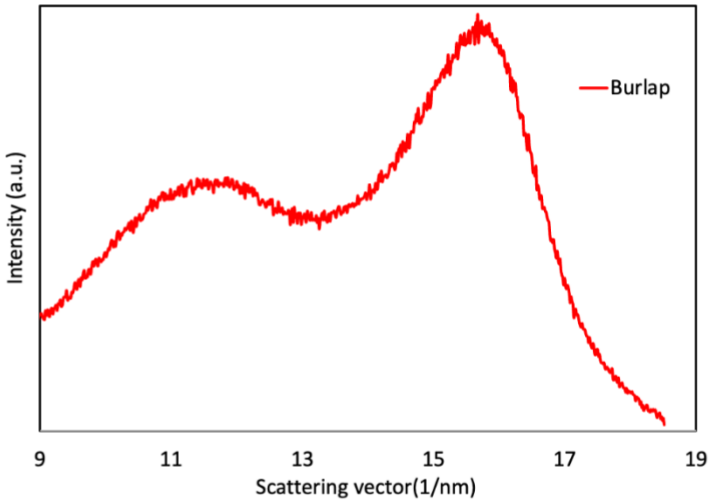

Figure S2. Wide-angle x-ray scattering (WAXS) patterns of burlap fibers showing crystalline reflections consistent with jute.

Setup for Immersion in Marine Coastal Environment

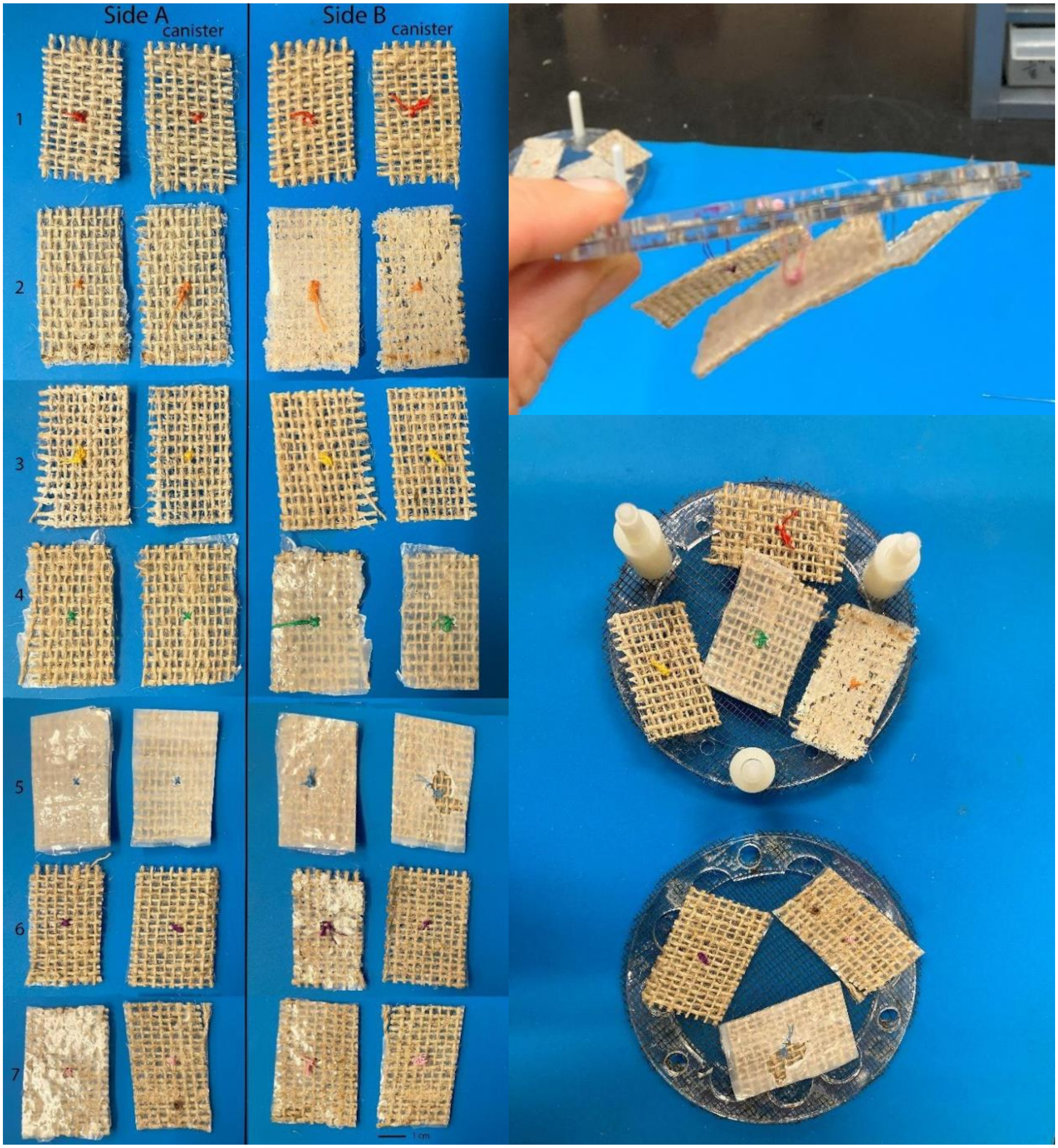

Figure S3. Photographs of the environmental testing setup. Burlap samples (coated and uncoated) were mounted in fish-exclusion canisters and submerged at ~5 m depth at the URI GSO dock. Images show the mounting configuration prior to deployment.

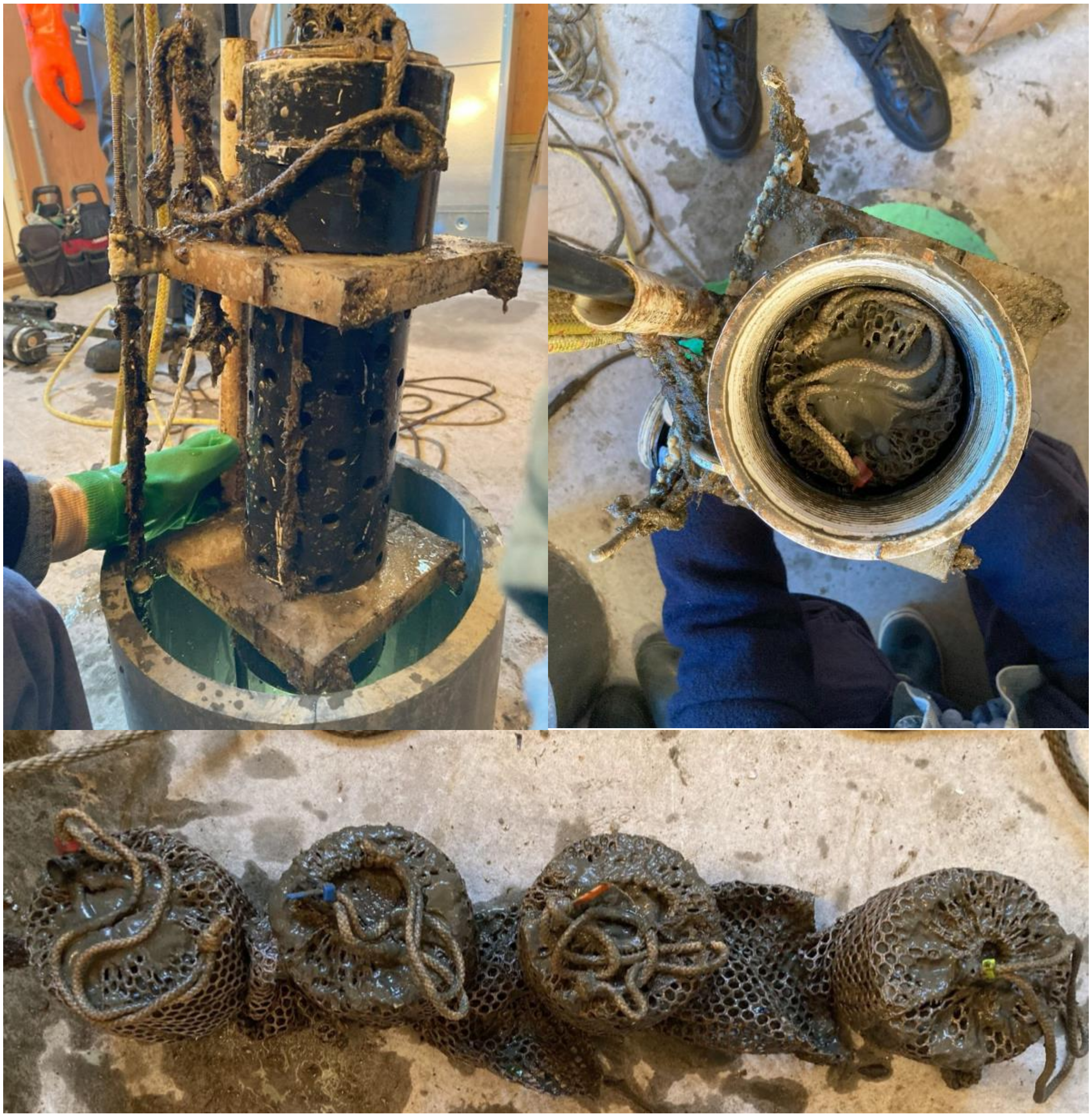

Figure S4. Example of monthly sample retrieval. The canister is pulled from the dock, opened, and individual samples are photographed to document progressive degradation during immersion. Photographs illustrate structural changes in coated and uncoated burlap over time.

GPC Fitting

Molecular weight of P1 was evaluated using gel permeation chromatography (GPC) calibrated with polystyrene (PS) standards and converted to P3HB values using the Mark–Houwink–Sakurada relationship:

$$K_{PHB}M_{PHB}^{\alpha} = K_{PS}M_{PS}^{\alpha} \tag{S1}$$

K and α constants for PS were taken from the *Polymer Handbook* [1]. For P3HB, three literature parameter sets (drawn from two independent references in the *Polymer Handbook*) were applied. Calculations were performed for each Gaussian-deconvoluted peak of the GPC trace. Results are summarized in Table S1.

Table S1. Number-average molecular weights (M_n) obtained for each Gaussian-deconvoluted GPC peak of P1 before and after 288 days of exposure. Values were first calculated using PS standards and then converted to PHB equivalents using three Mark–Houwink–Sakurada parameter sets.

			**M_n before**			**M_n after**		
	α	K x 10^3 (mL/g)	Peak 1	Peak 2	Peak 3	Peak 1	Peak 2	Peak 3
**PS**	0.794	7.2	5495	3950	1179	4597	2124	740
**P1-1**	0.78	11.8	3404	2433	711	2839	1294	442
**P1-2**	0.82	7.7	3853	2799	868	3242	1535	553
**P1-3**	0.76	16.6	2691	1906	539	2233	997	331

WAXS Fitting

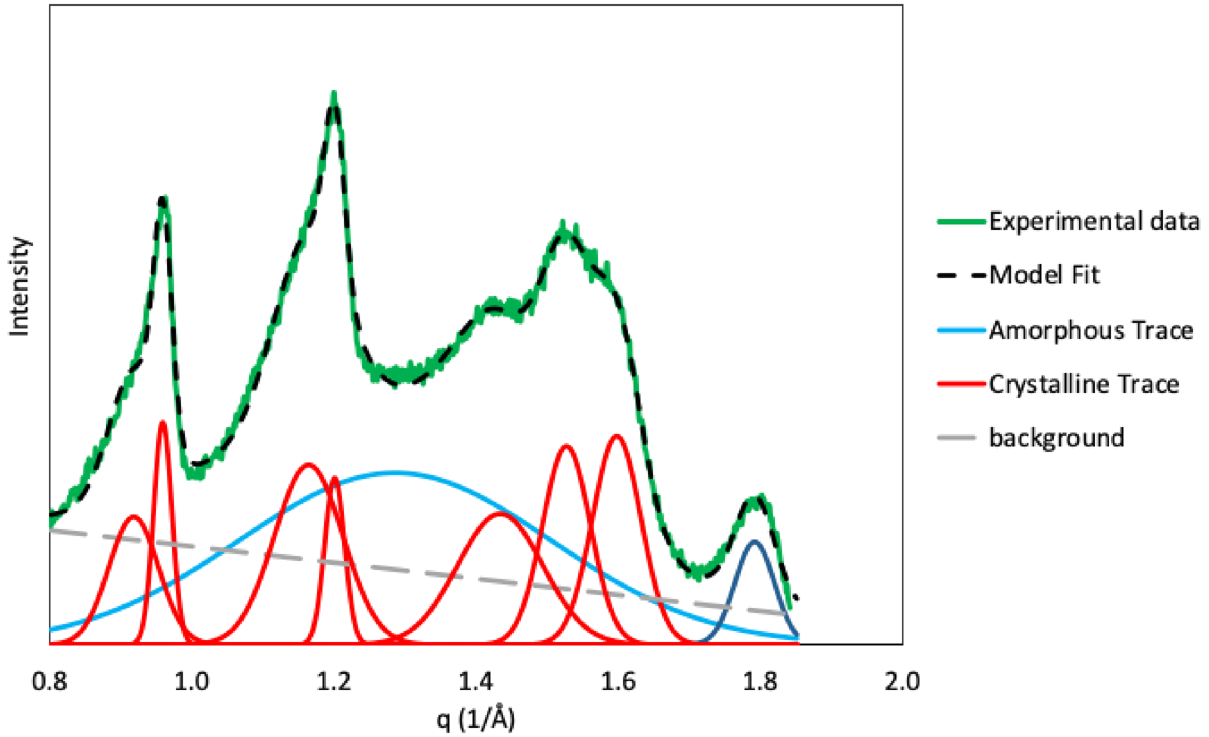

Figure S5. Representative WAXS curve fitting for PHA coatings. Crystalline and amorphous peaks were deconvoluted, and the degree of crystallinity (X_c) was calculated from integrated peak areas.

Table S2. Degree of crystallinity of polymer coatings (P1, P2, P3) determined from WAXS fits (see Figure S5). Values are reported separately for single-sided and double-sided coatings.

**Sample**		$\boldsymbol{X_c}$ (%)
**1-sided**	P1	58
	P2	50
	P3	37
**2-sided**	P2	52
	P3	42

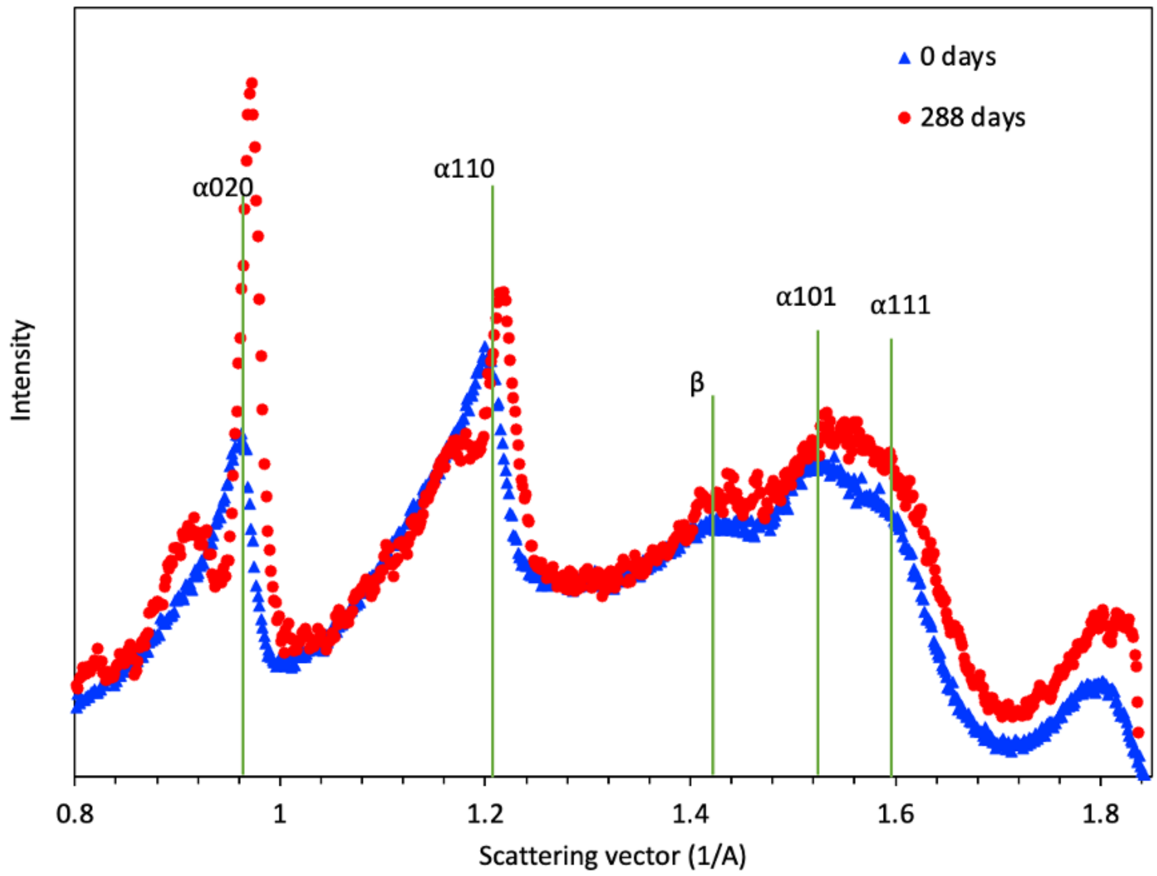

Figure S6. Identification of peaks in WAXS patterns of P1 at 0 days (blue) and after 288 days of immersion (red). Vertical markers denote α and β diffraction peaks. [2]

From WAXS data, approximately 20% of crystals are in β-form, which makes the average x_0 for the polymer:

$$x_0 = n_\alpha * x_\alpha + n_\beta * x_\beta \quad \text{(S2)}$$

From this calculation, we get effective x_0 value of 0.332 nm.

SAXS Analysis

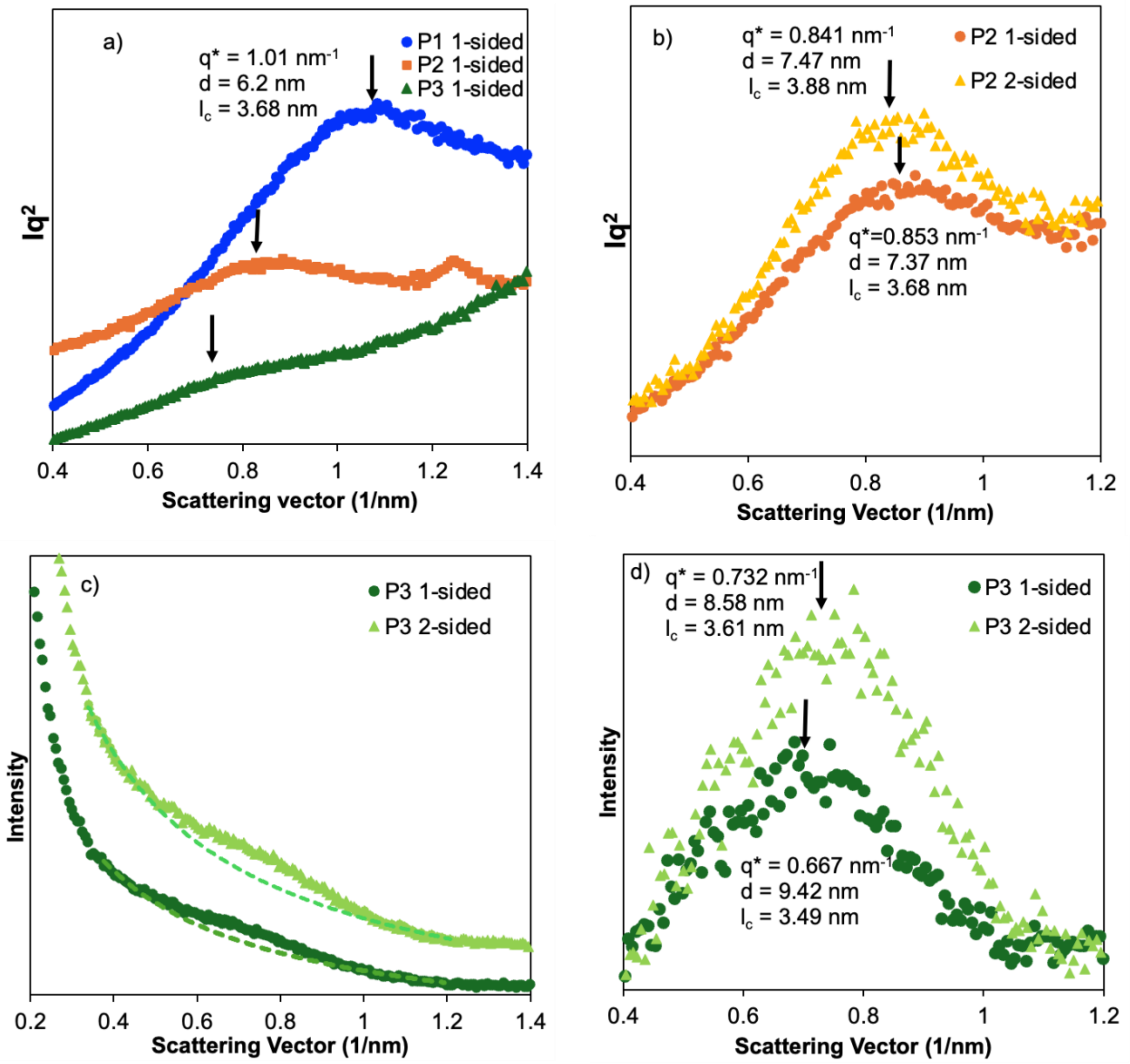

Figure S7. SAXS analysis of melt-pressed polymer samples at t = 0. (a) Plot of Iq^2 vs. q for P1, P2, and P3 single-sided melt-press coatings; although primary peak locations (q^*) and lamellar long period (d) differ, lamellar thicknesses (l_c) calculated according to $l_c = d * X_c$ are not significantly different (3.5-3.7 nm) between samples. (b) Plot of Iq^2 vs. q for P2 single-sided and double-sided melt-press coatings. (c) Raw SAXS intensity profiles and (d) background-subtracted SAXS profiles for P3 single-sided and double-sided melt-press coatings. Double-sided melt-press coatings have slightly greater l_c than single-sided coatings (≈ 0.1-0.2 nm) for both P2 and P3, but this difference is likely within measurement/analysis uncertainty and may not be significant.

References

1. Mark, J.E. and J.E. Mark, *Polymer data handbook*. 1999, New York: Oxford University Press. xi, 1018 pages : illustrations.
2. Phongtamrug, S. and K. Tashiro, *X-ray Crystal Structure Analysis of Poly(3-hydroxybutyrate) β-Form and the Proposition of a Mechanism of the Stress-Induced α-to-β Phase Transition.* Macromolecules, 2019. **52**(8): p. 2995-3009.
